# Monitoring weekly progress of front crawl swimmers using IMU-based performance evaluation goal metrics

**DOI:** 10.3389/fbioe.2022.910798

**Published:** 2022-08-08

**Authors:** Mahdi Hamidi Rad, Vincent Gremeaux, Fabien Massé, Farzin Dadashi, Kamiar Aminian

**Affiliations:** ^1^ Laboratory of Movement Analysis and Measurement, EPFL, Lausanne, Switzerland; ^2^ Institute of Sport Sciences, University of Lausanne, Lausanne, Switzerland; ^3^ Swiss Olympic Medical Center, Sport Medicine Unit, Division of Physical Medicine and Rehabilitation, Lausanne University Hospital, Lausanne, Switzerland; ^4^ Gait Up S.A, Lausanne, Switzerland; ^5^ Huma Therapeutics Ltd., London, United Kingdom

**Keywords:** sports biomechanics, swimming, IMU sensor, swimming phase, phase-based evaluation, swimmer progress

## Abstract

Technical evaluation of swimming performance is an essential factor in preparing elite swimmers for their competitions. Inertial measurement units (IMUs) have attracted much attention recently because they can provide coaches with a detailed analysis of swimmers’ performance during training. A coach can obtain a quantitative and objective evaluation from IMU. The purpose of this study was to validate the use of a new phase-based performance assessment with a single IMU worn on the sacrum during training sessions. Sixteen competitive swimmers performed five one-way front crawl trials at their maximum speed wearing an IMU on the sacrum. The coach recorded the lap time for each trial, as it remains the gold standard for swimmer’s performance in competition. The measurement was carried out once a week for 10 consecutive weeks to monitor the improvement in the swimmers’ performance. Meaningful progress was defined as a time decrease of at least 0.5 s over a 25 m lap. Using validated algorithms, we estimated five goal metrics from the IMU signals representing the swimmer’s performance in the swimming phases (wall push-off, glide, stroke preparation, free-swimming) and in the entire lap. The results showed that the goal metrics for free-swimming phase and the entire lap predicted the swimmer’s progress well (e.g., accuracy, precision, sensitivity, and specificity of 0.91, 0.89, 0.94, and 0.95 for the lap goal metric, respectively). As the goal metrics for initial phases (wall push-off, glide, stroke preparation) achieved high precision and specificity (≥0.79) in progress detection, the coach can use them for swimmers with satisfactory free-swimming phase performance and make further improvements in initial phases. Changes in the values of the goal metrics have been shown to be correlated with changes in lap time when there is meaningful progress. The results of this study show that goal metrics provided by the phase-based performance evaluation with a single IMU can help monitoring swimming progress. Average velocity of the lap can replace traditional lap time measurement, while phase-based goal metrics provide more information about the swimmer’s performance in each phase. This evaluation can help the coach quantitatively monitor the swimmer’s performance and train them more efficiently.

## Introduction

Swimming coaches aim to improve the performance of swimmers in intensive training sessions and prepare them for competition. Depending on the event, the swimmer completes multiple sets, each of which includes several swimming phases: a dive or wall push-off, a glide underwater, a stroke preparation, free-swimming to the end, and a turn to continue the next round with the same sequence of phases. Coaches should focus on each phase because a flawless performance by the swimmer in every phase is necessary to win (Mooney et al., 2016b). They mostly rely on observation and personal experience to monitor and evaluate a swimmer’s performance. A coach expects swimmers to improve their performance by 1%–10% during a training season, depending on swimmer’s level (Zacca et al., 2020; Ferreira et al., 2021), and usually tracks this progress by measuring lap time over different swimming distances (most commonly 400 m, as it is used to evaluate the swimmer’s aerobic performance). However, lap time can only reflect the swimmer’s overall progress and not their phase-based performance. The use of biomechanical parameters such as stroke rate, stroke length, and stroke index (product of average velocity and stroke length) (Morais et al., 2013) or body composition (Thng et al., 2022) are other methods proposed by researchers to track swimmer’s progress.

The complexity of extracting performance-related parameters has led coaches to use technological tools to obtain an objective and quantitative analysis (Payton and Adrian Burden, 2017). Swimming coaches use a variety of analysis systems such as 2D and 3D cameras (Mooney et al., 2015), inertial measurement unit (IMUs) (Guignard et al., 2017), or physiological parameters such as heart rate (Crowcroft et al., 2017), or lactate monitors (Smith et al., 2002) to investigate the technical aspects of swimming. Although video-based systems are still the gold standard for swimming analysis, they generally suffer from several limitations in aquatic environments, such as cumbersome installation and calibration, water splashes and reflections, or limited recording volume (Callaway et al., 2010). As a result, there is still a need in the coaching community for supportive analysis systems (Mooney et al., 2016a). Improvements in the accuracy, scalability, and cost of Micro-electromechanical systems (MEMS) have led to IMUs becoming a credible option for swimmer motion tracking, as they can provide quick and easy-to-use feedback on detailed performance-related metrics (Ramos Félix et al., 2019).

Several studies have investigated the analysis of swimming with IMUs by extracting kinematic parameters in different phases and techniques such as stroke rate and stroke count (Davey and James, 2008), instantaneous velocity (Dadashi et al., 2012), tumble turn spatio-temporal parameters (Slawson et al., 2012) or wall push-off maximum velocity (Stamm, 2013). Although these studies have demonstrated the application of IMUs for swimming analysis, they have not related the obtained kinematic parameters to the swimmer’s performance-related metrics. In our previous study, we used IMUs to automatically segment each swimming lap into wall push-off (*Push*), glide (*Glid*), stroke preparation (*StPr*), free-swimming (*Swim*), and turn phases (Hamidi Rad et al., 2021b). The algorithms developed in this study take a macro-micro approach by swimming bouts detection, lap separation, and swimming style identification at the macro level, and then divide each lap into phases by detecting spatio-temporal events on IMU acceleration and angular velocity data at the micro level. Subsequently, a variety of kinematic parameters were extracted from each phase and used to estimate phase-based goal metrics (*Push* maximum velocity, *Glid* end velocity, *StPr* average velocity, *Swim* average velocity and lap average velocity) for the swimmer’s performance evaluation (Hamidi Rad et al., 2021a), indicating how well the swimmer performed the corresponding phase. However, to fully utilize the IMU sensor for training, assessing the sensitivity of IMU-based goal metrics to performance progress is of utmost importance.

Therefore, the main objective of this study was to validate the use of IMU-based goal metrics to monitor swimming performance during training sessions. Using the macro-micro approach to swimming analysis to separate the swimming phases (*Push*, *Glid*, *StPr*, and *Swim*) and the phase-based performance assessment on sacrum IMU, we estimated the goal metrics of each phase. We then analyzed the sensitivity of goal metrics in relation to the swimmer’s progress across multiple training sessions. We assumed that 1) lap time is the most important representative of performance level and can be used to define meaningful progress, and 2) the goal metrics change in association with lap time when the swimmer makes meaningful progress.

## Materials and methods

### Measurement setup and protocol

Sixteen competitive swimmers from a swimming team participated in this study, and their characteristics are shown in Table 1. A waterproof band (Tegaderm, 3M Co., United States) was used to attach an IMU (Physilog^®^ IV, GaitUp, CH.) to the swimmer’s lower back on sacrum bone. The sensor recorded 3D angular velocity (±2000°/s) and 3D accelerometer (±16 g) at a sampling rate of 500 Hz. After installation of the sensor, functional calibration was performed with simple out-of-water movements (upright standing and squatting) to make the data independent of the sensor exact position on swimmer’s sacrum (Dadashi et al., 2013).

**TABLE 1:** Statistics of the swimmers. The values are presented as mean ± standard deviation.

Male	Female	Age (yrs)	Height (cm)	Weight (kg)	50 m Front crawl record (s)
9	7	14.6 ± 0.8	171.6 ± 6.9	55.9 ± 10.1	28.60 ± 2.04

**FIGURE 1:**
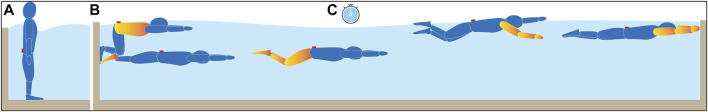
Measurement protocol with IMU (red box) attached to the sacrum. After functional calibration, the swimmer starts in the water with an upright posture **(A)** and performs all swimming phases at maximum speed while swimming to the other side in front crawl **(B)**. The coach records the lap time with a stopwatch during each lap **(C)**.

After a brief warm-up, swimmers were asked to swim five times one swimming pool length (one lap) in the same direction at maximum velocity, beginning with a 5-s upright stance before wall push-off in the water (Figures 1A). During a full lap, the swimmer went through all swimming phases so that we could analyze the goal metrics of each phase (Figures 1B). The coach recorded the lap time of all swimmers with a stopwatch during each attempt (Figures 1C). Each swimmer had 5 min rest between trials to avoid fatigue. To track the swimmers’ progress, the same measurement was repeated once a week for ten sessions. Prior to participation, the measurement procedure was explained to each swimmer and they provided written informed consent. The measurement protocol of this study was approved by the EPFL Human Research Ethics Committee (HREC, No. 050/2018).

### Lap segmentation and phase-based performance evaluation

First, swimming bouts and laps were determined during each training session according to the validated algorithms of our macro-micro approach and then divided into four swimming phases of *Push*, *Glid*, *StPr* and *Swim* (Hamidi Rad et al., 2021b). *Push* phase begins with the forward movement of the swimmer’s trunk and ends when the feet leave the wall. *Glid* phase lasts until the beginning of the dolphin kicks in front crawl style. *StPr* phase is the next phase that ends with the first arm stroke, which is the beginning of the *Swim* phase, and *Swim* phase ends when the swimmer’s hand touches the wall. The method uses motion biomechanics to identify the events corresponding to the beginning and end of each phase for lap segmentation. Subsequently, based on our phase-based performance evaluation method (Hamidi Rad et al., 2021a), a set of spatio-temporal parameters reflecting various aspects of swimmer’s performance were extracted from each phase. These parameters are categorized as propulsion, posture, efficiency and duration/rate to represent the most important aspects of performance. They were fed into *LASSO* (Least Absolute Shrinkage and Selection Operator) regression models to estimate five phase-based goal metrics that quantify the performance within each phase: *Push* maximum velocity, *Glid* end velocity, *StPr* average velocity, *Swim* average velocity, and lap average velocity respectively for phases of push, glide, stroke preparation, swim and the entire lap. These goal metrics were tracked during the measurements to assess their sensitivity to swimmer progress during weeks of training.

### Sensitivity analysis

Sensitivity analysis was performed to assess how phase-based goal metrics react to swimmer’s progress in two steps. In the first step, we considered all sessions of each swimmer with a significant change in lap time, as lap time is considered representative of swimming performance (Robertson et al., 2009). Using the data from the weekly measurements, we compared the swimmer’s performance in each session to other sessions to find significant progress. According to the measurement protocol, five values (for each goal metric and for lap time) are obtained from each participant per session. Because the sample size for comparison between two sessions is small, we used Cliff’s Delta (*d*) effect size analysis as a nonparametric method (Macbeth et al., 2011). This method allowed us to determine whether the achieved lap times and goal metrics differed significantly from one session to another. Each comparison set is assigned an effect size value to quantify the change (Eq. 1).

$$d=\frac{\#\left(x_{i}>x_{j}\right)-\#\left(x_{i}<x_{j}\right)}{n_{1}n_{2}}$$ (1)

Where the cardinality symbol *#* indicates counting, $x_{i}$ and $x_{j}$ are the lap time or goal metric values of sessions *i* and *j*, respectively. $n_{1}$ and $n_{2}$ are the sizes of the two data sets, both equal to five in our study (i.e., the number of laps). The value of *d* estimates the probability that a value selected from the *i*th session is greater than a value selected from the *j*th session, minus the inverse probability. This can be referred to as a measure of dominance, indicating the degree of overlap between values from two test sessions. The *d* value is generally within the closed interval of [-1, +1] indicating the degree of overlap between the values from two sessions (effect size of +1.0 or −1.0 for no overlap and 0 for complete overlap). The effect size is considered significant if the confidence interval (*CI*) does not include zero. The upper and lower bounds of the asymmetric *CI* (range of $\delta_{lower}\;$ to $\;\delta_{higher}$ ) for Cliff’s *d* are constructed based on Eqs 2–4 as a more robust and conservative method (Feng and Cliff, 2004). $t_{\text{α}/2}$ is the critical value of the t-distribution for the corresponding confidence level.

$$d_{i}=\frac{\#\left(x_{i}>x_{j}\right)-\;\#\left(x_{i}<x_{j}\right)}{n_{1}},\;d_{j}=\frac{\#\left(x_{j}>x_{i}\right)-\;\#\left(x_{j}<x_{i}\right)}{n_{2}}$$ (2)

$$s_{d}^{2}=\frac{n_{1}^{2}\sum_{i=1}^{n_{1}}{\left(d_{i}-d\right)}^{2}+n_{2}^{2}\sum_{j=1}^{n_{2}}{\left(d_{j}-d\right)}^{2}+n_{2}^{2}\sum_{i=1}^{n_{1}}\sum_{j=1}^{n_{2}}{\left(d_{ij}-d\right)}^{2}}{n_{1}n_{2}\left(n_{1}-1\right)\left(n_{2}-1\right)}$$ (3)

$$\delta_{lower},\delta_{higher}=\frac{d-\;d^{3}\pm t_{\alpha /2}s_{d}{\left(1-2d^{2}+d^{4}+t_{\alpha /2}^{2}s_{d}^{2}\right)}^{1/2}}{1-d^{2}+t_{\alpha /2}^{2}s_{d}^{2}}$$ (4)

Thus, the effect size values along with the *CI* ranges were calculated for comparing the five values of goal metrics or lap time between every two sessions using Eqs 1–4 and the significant pairs were separated. However, all significant changes in lap time should not be considered as meaningful progress. This is because the lap time value itself is subject to recording errors (using the stopwatch). Based on the training plan, the coach expected to see real progress in the swimmers after at least 3 weeks of training. Therefore, a meaningful lap time change (*MLTC*) was defined as the minimum threshold for meaningful progress. It is indeed similar to the concept of smallest worthwhile enhancement which is defined for competitions to estimate the minimum amount of improvement that is beneficial for athletes to win a race (Hopkins et al., 1999). However, we tend to compare swimmers only with themselves and not with others in training sessions. So we calculated the median lap time of comparisons that were 3 weeks apart (session 1 and session 4, session 2 and session 5, etc.). *MLTC* is then calculated by taking the average of the differences of all these comparison pairs over all swimmers.

In the second step of the sensitivity analysis, among all significant differences identified in step one between test sessions, only those with a median change more than *MLTC* were retained as meaningful progress. The entire process of the two steps for detecting significant pairs and then selecting the pairs with meaningful progress is explained by the following pseudocode, where *m* and *n* are two different session numbers that vary across all sessions with two loops and *LT*_*i,j*_ is *i*th lap time of *j*th session.


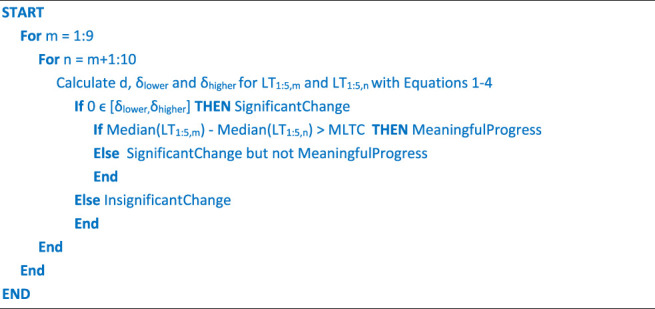


After obtaining all the pairs with meaningful progress, the relationship between changes of goal metrics and changes in lap time was examined for these pairs to analyze the sensitivity of goal metrics to progress by answering three questions:

(i) “Do the goal metrics predict meaningful progress, as does lap time?”
(ii) “How well do the goal metrics represent the swimmer’s performance compared to the lap time?”
(iii) “What is the contribution share of each goal metric to swimming progress?”

To answer the first question, we analyzed the correspondence between progress detection by each goal metric and lap time. For each pair of sessions, we calculated whether the change (i.e., improvement) in the values of goal metrics was significant (i.e., true) or not significant (i.e., false) and then compared it to the meaningfulness of the change in lap time. The performance of goal metrics in predicting meaningful progress (i.e., a significant lap time more than *MLTC*) was assessed using the following association rules:

• True positive (*TP*): goal metric shows a significant change when there is a meaningful progress.
• True negative (*TN*): no significant change is observed with goal metric when there is no meaningful progress.
• False positive (*FP*): no meaningful progress, while the goal metric changes significantly.
• False negative (*FN*): meaningful progress, while the goal metric does not show significant change.

The values for accuracy, precision, specificity, and sensitivity to predict meaningful progress are calculated for each goal metric using Eqs 5–8.

$$Accuracy\;=\frac{TP+TN}{TP+TN+FP+FN}$$ (5)

$$Precision=\frac{TP}{TP+FP}$$ (6)

$$Sensitivity=\frac{TP}{TP+FN}$$ (7)

$$Specificity=\frac{TN}{TN+FP}$$ (8)

To answer the second question, how well the goal metrics represent swimming performance, effect size values were estimated for each significant change in the goal metric and compared to the effect size of lap time if there was a meaningful progress. The third question is about the relationship between the magnitude of change in each goal metric (i.e., change of *Push* maximum velocity (Δ*Push*), *Glid* end velocity (Δ*Glid*), *StPr* average velocity (Δ*StPr*), *Swim* average velocity (Δ*Swim*), and lap average velocity (Δ*Lap*)) and the change in lap time (Δ*LapTime*) when there is a meaningful progress. This analysis is performed by calculating the Pearson correlation (Benesty et al., 2009) between the changes in goal metrics and lap time values.

## Results

**FIGURE 2:**
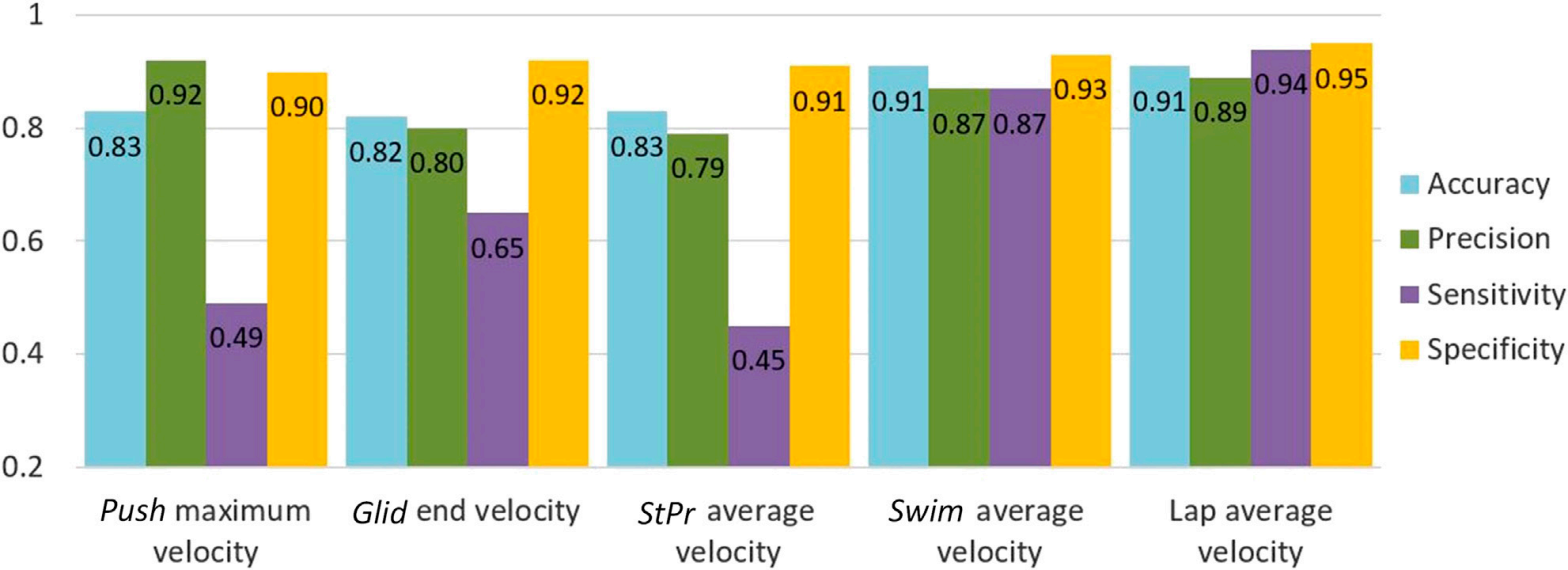
Accuracy, precision, sensitivity and specificity of goal metrics for detecting a meaningful progress (lap time change).

A post-hoc sample size analysis was performed (Jones et al., 2003) considering the lowest acceptable sensitivity and specificity of 0.90 and 0.80, respectively, with a confidence interval of 90%, resulting in a sample size of 107 for this study. This means that at least this number of meaningful comparisons are needed to make a valid comparison between the change in goal metrics and the change in lap time. During the ten measurement sessions, there were seven absences due to swimmers being unavailable, and a total of 750 swimming laps were recorded. Each swimmer is compared to themselves during all measurement sessions, and 642 comparisons were made for all swimmers. 272 of the comparisons showed statistically significant progress (based on Cliff’s delta analysis at a 95% confidence level). The accuracy, precision, sensitivity, and specificity of each of the goal metrics used to detect this significant change in lap time (i.e., the first step of the sensitivity analysis) can be found in the Supplementary Figure SA1. Next, comparison of sessions 3 weeks apart for the second step of the analysis yielded an *MLTC* value of 0.5 ± 0.2 *s*, resulting in 122 pairs of sessions with meaningful progress which is higher that the sample size. Each swimmer showed at least four comparison pairs with meaningful progress. The slower the swimmer was during the first test session (higher median of lap time), the higher the number of comparison pairs with meaningful progress (significant correlation coefficient of 0.70), because the swimmers who swim relatively slower have more room for performance improvement. The accuracy, precision, sensitivity, and specificity of each goal metric for detecting meaningful progress are shown in Figure 2.

Among the five metrics, lap and *Swim* average velocity achieved the highest values for accuracy, sensitivity, precision, and specificity (≥0.87). For the three metrics related to the initial phases of *Push*, *Glid* and *StPr*, precision and specificity were relatively high (≥0.79), whereas sensitivity was low (0.45–0.65). For the comparisons in which both meaningful progress in lap time was detected and the goal metric was significant, the effect size values and confidence interval were calculated (Table 2). Comparison of the effect size values for each goal metric and lap time shows lap average velocity and *Swim* average velocity are the best ones for progress detection (difference of 0.04 between effect size values). However, the other three goal metrics achieved lower effect size values than lap time.

**TABLE 2:** Effect size and confidence interval of all goal metrics and lap time for the comparisons with both meaningful progress and significant goal metric change.

Goal metric		*Push* maximum velocity	*Glid* end velocity	*StPr* average velocity	*Swim* average velocity	*Lap* average velocity
Effect size [CI]	Goal metric	0.67 [0.26, 0.85]	0.78 [0.30, 0.90]	0.75 [0.26, 0.89]	0.92 [0.25, 0.96]	0.93 [0.27, 0.97]
	Lap time	0.96 [0.25, 0.98]				

**FIGURE 3:**
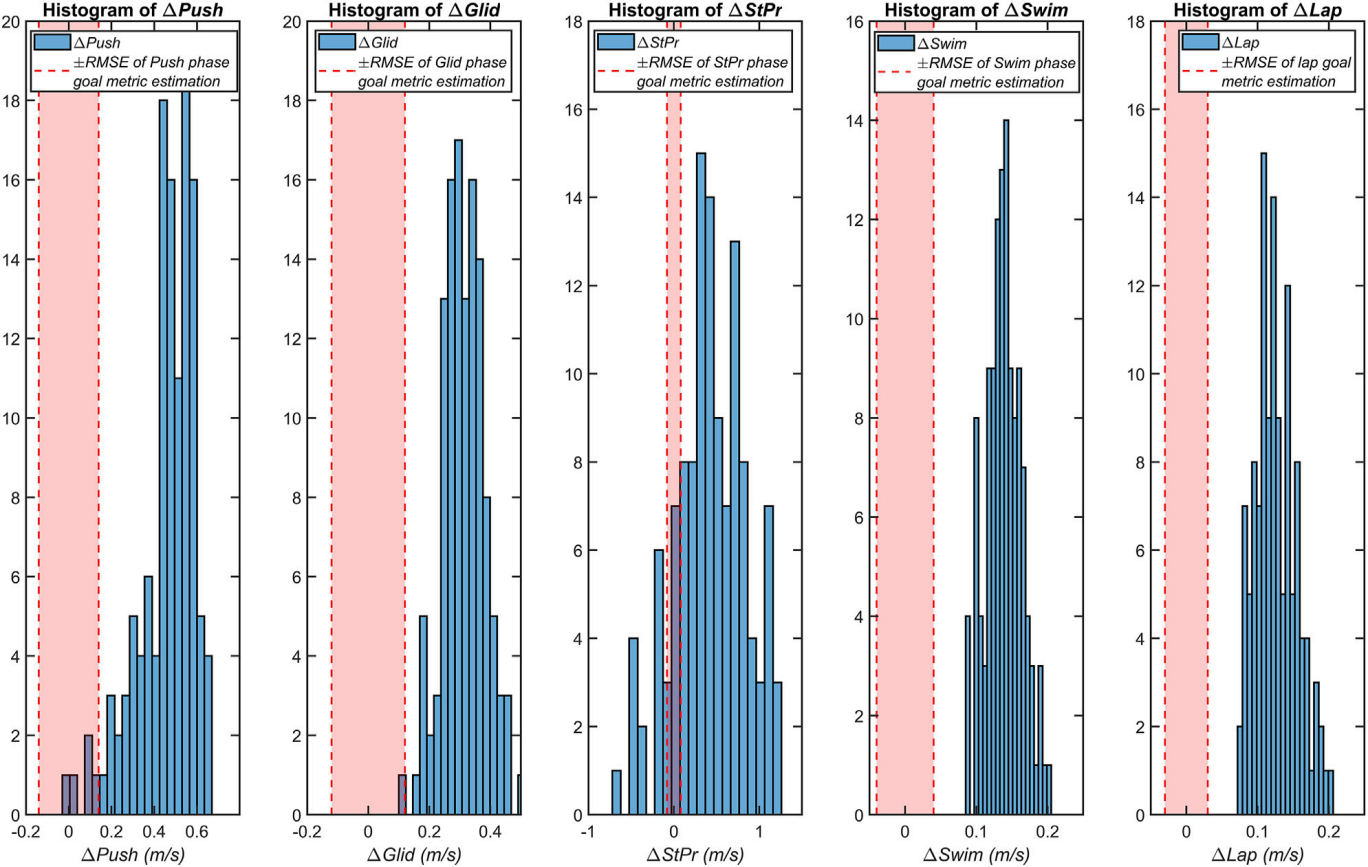
Histograms of changes in the five IMU goal metrics (Δ*Push,* Δ*Glid,* Δ*StPr,* Δ*Swim,* and Δ*Lap*) for the comparisons with meaningful progress. The estimation *RMSE* range of each goal metric is displayed with red dashed lines.

The final set of results addresses the correlation analysis between the magnitude of changes in the goal metrics (Δ*Push*, Δ*Glid*, Δ*StPr*, Δ*Swim*, and Δ*Lap*) and in lap time (Δ*LapTime*) across all comparisons with meaningful progress. Histograms of the changes in the goal metrics are displayed in Figure 3. The root mean squared error (*RMSE*) for the estimation of each goal metric is extracted from our previous study (Hamidi Rad et al., 2021a) and shown specifically for each goal metric in vertical red lines in Figure 3. The delta values lying inside the range of *RMSE* (±*RMSE* range) are too small to be valid as they might happen due the model errors and should be removed. After removing the invalid delta values for each goal metric, we analyzed the contribution of each metric to the progress of swimming performance. Table 3 shows the average, standard deviation, and range for the changes in the goal metrics, as well as their correlation coefficient (*r*) with Δ*LapTime*. Of the five goal metrics, Δ*StPr* shows the highest standard deviation (0.40 m*/s*). With the exception of Δ*Push*, the change values of all goal metrics were significantly correlated with Δ*LapTime*, however with weak correlation coefficients (Table 3).

**TABLE 3:** Average, standard deviation, and range of each goal metric change and its correlation coefficient (*r*) with Δ*LapTime* for all meaningful progress comparisons. The change values that are below *RMSE* of each goal metric are removed.

Goal metric change	Δ*Push*	Δ*Glid*	Δ*StPr*	Δ*Swim*	Δ*Lap*
Average (*m/s*)	0.49	0.33	0.50	0.14	0.13
Standard deviation (*m/*s)	0.09	0.06	0.40	0.02	0.03
Range (*m/s*)	0.52	0.44	1.89	0.16	0.17
Correlation coefficient (*r*) with Δ*LapTime*	−0.04	−0.21^**^	−0.17^**^	−0.29^***^	−0.31^***^

*p-value < 0.05, ** *p*-value < 0.01, *** *p*-value < 0.001.

## Discussion

In this study, a single IMU, worn on sacrum, was used to identify the four major phases of a swimming lap and calculate a performance-based goal metric for each of these phases and the entire lap. These goal metrics were then used to follow the swimmers’ progress over ten training sessions. The results obtained confirmed our hypothesis of association between the phase-based goal metrics and swimmers’ progress, but with varying sensitivity and degree of association in each phase.

As shown in Figure 2, lap average velocity and *Swim* average velocity achieved the highest accuracy, precision, sensitivity, and specificity (≥0.87) among all goal metrics to predict meaningful progress. Because lap time is used as a representative of performance, lap average velocity was expected to be highly associated with it. This goal metric could replace traditional lap time because it is not affected by human recording error. Furthermore, since the *Swim* phase is the longest phase of a lap, it should contribute more to lap time compared to other phases. Although the sensitivity of *Push* maximum velocity, *Glid* end velocity, and *StPr* average velocity are low, their specificity and precision are either at or above 0.80. Considering Eqs 6–8, the high specificity and precision is mainly due to a low number of false positives. It can be concluded that the three initial goal metrics are less good at detecting meaningful progress than the other two metrics. However, when they do detect progress, it is correct, indicating that they are relevant to progress assessment despite their low sensitivity.

Compared with similar results using goal metrics to detect significant (and not meaningful defined by *MLTC*) progress shown in Supplementary Figure SA1, using meaningful progress improved the results. The accuracy, precision, sensitivity, and specificity of all five goal metrics for detecting significant progress were lower because the procedure was affected by the lap time recording error. However, the sensitivity of the goal metrics for the initial phase remained low for the same reason. Overall, it appears that all phases are important for improving overall performance and progress is the result of mastering all phases of swimming. The coach can use the three metrics of the initial phases to provide an additional quantitative assessment. However, this argument does not apply in reverse, and a change in lap time is not essentially the result of better performance in the initial phases. It increases the number of false negatives and lowers the sensitivity of the initial phases goal metrics to overall progress.

In terms of effect sizes and confidence interval ranges, Table 2 shows that the effect size values of the goal metrics for lap average velocity and *Swim* average velocity are closest to the effect size of lap time, such that these two metrics are as strong as lap time in indicating progress. However, the effect size values of the goal metrics *Push* maximum velocity, *Glid* end velocity, and *StPr* average velocity are lower than lap time because they cannot represent the overall performance of the swimmers as well as lap time. It can be argued that if the swimmer is not making more progress in the *Swim* phase, there is still room for improvement in the initial phases and the coach should focus on these goal metrics to make further progress.

Figure 3 shows that among the five changes in the goal metric, only Δ*StPr* has worsened in some cases, while there is a meaningful progress on lap performance (negative values of the histogram). Due to the coaching strategy at this period of the season, the coach did not emphasize working on this phase for the swimmers with weak performances, and asked them to focus on other phases to compensate. Most of the change values of all goal metrics are outside the range of the *RMSE* of the goal metric estimation. The correlation coefficients of the changes of all goal metrics with Δ*LapTime* are weak (<0.4) (Table 3). Since the change values of the goal metrics are reliable after removing the samples lying inside the ±*RMSE* range (Figure 3), the main reason for the weak correlation is the error in recording the lap time, since it is recorded by the coach with a handheld stopwatch, while this analysis requires a more precise method. However, since the correlations are significant, we can conclude that improving goal metrics contributes to swimmer’s progress and the coach should use all of these metrics in the training sessions.

In order to obtain a larger, more varied data set, both male and female swimmers were used to generate our results, and comparison based on individual differences is beyond the scope of this study. For technical reasons, only front crawl technique is examined here. However, based on our previous research (Hamidi Rad et al., 2021a), similar goal metrics can be extracted from other main swimming techniques (backstroke, butterfly, and breaststroke) to perform the same study. The lap time was recorded using stopwatch which is prone to human error and using more precise measurement methods such as cameras can increase the quality of this analysis. Since we had only one-way laps in the measurements, the turn phase was not evaluated in this study. The number of lap repetitions per swimmer was limited to five to avoid a fatigue effect that could affect the assessment of progress. However, collection of a larger data set would be required to perform a more powerful statistical analysis.

This study shows that the goal metrics calculated from a single sacrum IMU can provide valuable information about performance in different swimming phases. Coaches can forgo measuring lap time with a stopwatch and use the goal metric for lap average velocity, which can be automatically estimated based on IMU as a substitute for traditional lap timing. They can then focus on the goal metric for each phase to get a more detailed analysis of the swimmer’s performance. Compared to other studies monitoring swimmers’ performance that focused mainly on either overall performance or free-swimming phase parameters (Morais et al., 2013, 2015), our proposed goal metrics allow the coach to track swimming performance in each phase separately. Furthermore, tracking progress using conventional methods such as video-based systems or heart rate and lactate monitors is very time-consuming and only possible at selected times during a season (Ferreira et al., 2021), whereas IMUs have the least impact on swimmers’ training and can be used on a daily basis.

The dominance of coaching philosophy and qualitative analysis in training sessions invariably leads to subjective, inaccurate assessments (Mooney et al., 2016a). Therefore, providing phase-based goal metrics serves as an assistant to the coach, allowing him or her to quantitatively monitor each swimming phase and track a swimmer’s progress during training sessions. Using this information, the coach can customize training strategies for each swimmer, which usually takes a lot of time and effort. Although wearables induce more drag on the swimmer’s body (Magalhaes et al., 2015), they require an extremely small amount of preparation and analysis from the coach to provide personalized feedback. The coach can access performance evaluation reports for the entire team after each training session and plan further training for each swimmer based on their phase-specific progress.

## Conclusion

By using IMU based goal metrics to monitor the performance of a team of swimmers, we have demonstrated the possibility of objective evaluation of swimmers’ progress during training sessions. Of the goal metrics considered in this study, lap average velocity and Swim average velocity had the highest accuracy, precision, sensitivity, and specificity (≥0.87) to predict swimmers’ progress. The goal metrics related to *Push*, *Glid* and *StPr* achieved high specificity and precision (≥0.79) for progress, confirming the role of initial phases in overall swimming performance. Lap average velocity and *Swim* average velocity are as sensitive as lap time to swimming progress and can be used as precise performance-related indicators. Other goal metrics provide additional quantitative information about the swimmer’s phase-related performance that is not available in traditional coaching approaches. It is illustrated that the value of changes in goal metrics also correlates with swimmer progress. In summary, the coach can use the phase-based report to obtain a comprehensive view of the swimmer’s performance. This study opens new training horizons in swimming by providing objective feedback based on goal metrics and analyzing the effects of feedback on the swimmer’s performance.

## Data Availability

The raw data supporting the conclusions of this article will be made available by the authors, without undue reservation.
