# Supplementary material for: Monitoring weekly progress of front crawl swimmers using IMU-based performance evaluation goal metrics
Source: Front Bioeng Biotechnol. 2022 Aug 8;10:910798. doi: 10.3389/fbioe.2022.910798 (PMC9393393; doi:10.3389/fbioe.2022.910798)
Supplement: Supplementary file 1 [file DataSheet1.docx]

Supplementary Material

# Sensitivity analysis with significant progress

To find meaningful progress, we defined meaningful lap time change (*MLTC*) based on the hypothesis that significant lap time change does not necessarily represent meaningful performance change and could be transient. To compare the results before and after using *MLTC*, Figure A1 presents the accuracy, precision, sensitivity, and specificity of each goal metric for detecting a significant change in lap time (based on Cliff's delta confidence interval only).


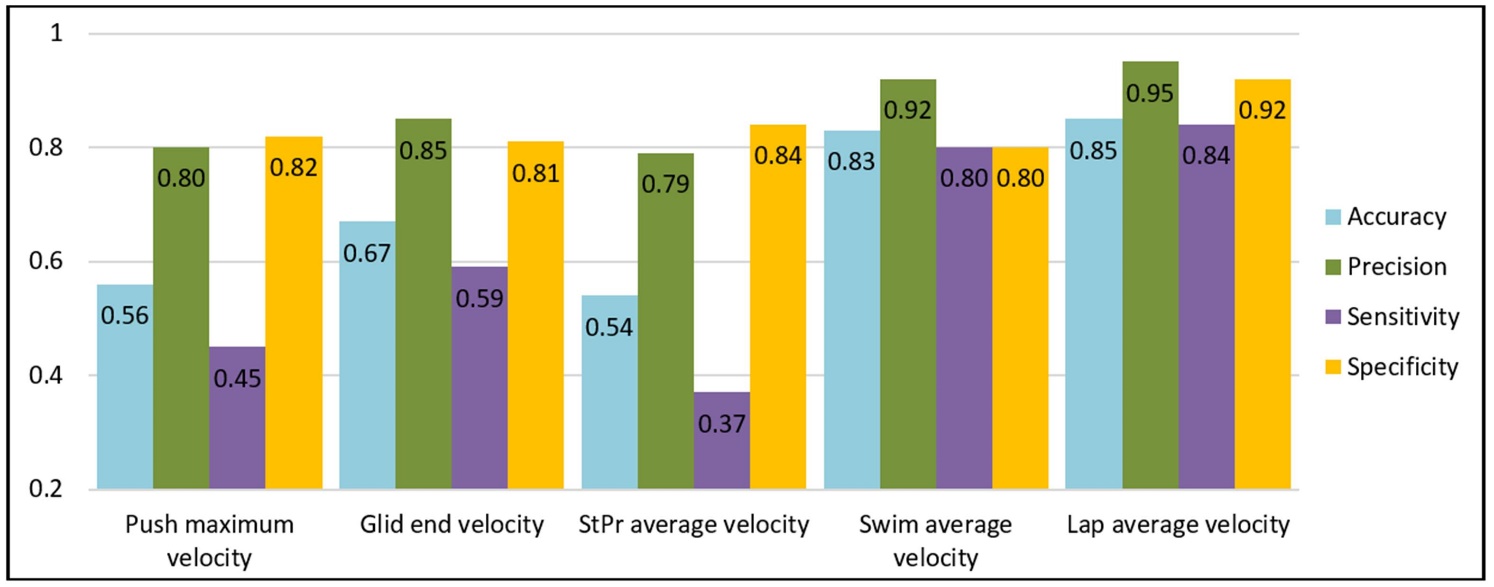


Figure A1 - accuracy, precision, sensitivity and specificity of goal metrics for detecting a significant progress (lap time change).

# Glossary of terms

Here if the table of glossary of all the terms used in this research.

Table A1 – Table of glossary

| **Term** | **Definition** |
| --- | --- |
| *IMU* | Inertial measurement unit |
| *LASSO* | least absolute shrinkage and selection operator |
| *RMSE* | Root mean square error |
| *Push* | Wall push-off phase |
| *Glid* | Glide phase |
| *StPr* | Strokes preparation phase |
| *Swim* | Swimming phase |
| *d* | Cliff's Delta effect size |
| *CI* | Confidence interval |
| *MLTC* | Meaningful lap time change |
| *TP* | True positive |
| *TN* | True negative |
| *FP* | False positive |
| *FN* | False negative |
| Δ*Push* | Change in push maximum velocity |
| Δ*Glid* | Change in glide end velocity |
| Δ*StPr* | Change in strokes preparation average velocity |
| Δ*Swim* | Change in swim average velocity |
| Δ*Lap* | Change in lap average velocity |
| Δ*LapTime* | Change in lap time |
